# Blinding Measured: A Systematic Review of Randomized Controlled Trials of Acupuncture

**DOI:** 10.1155/2013/708251

**Published:** 2013-03-03

**Authors:** Alex Moroz, Brian Freed, Laura Tiedemann, Heejung Bang, Melanie Howell, Jongbae J. Park

**Affiliations:** ^1^The Center for Musculoskeletal Care and Department of Rehabilitation Medicine, New York University School of Medicine, 333 East 38th Street, 5th Floor, Room 5-103, New York, NY 10016, USA; ^2^Department of Neuroscience, Brown University, Providence, RI 02912, USA; ^3^Division of Biostatistics, Department of Public Health Sciences, University of California-Davis, Davis, CA 95616, USA; ^4^Asian Medicine and Acupuncture Research, Department of Physical Medicine and Rehabilitation, School of Medicine, University of North Carolina-Chapel Hill, Chapel Hill, NC 27514, USA; ^5^Orofacial Pain Program, Regional Center for Neurosensory Disorders, School of Dentistry, University of North Carolina-Chapel Hill, Chapel Hill, NC 27599, USA

## Abstract

*Background.* There is no agreement among researchers on viable controls for acupuncture treatment, and the assessment of the effectiveness of blinding and its interpretation is rare. *Purpose.* To systematically assess the effectiveness of blinding (EOB) in reported acupuncture trials; to explore results of RCTs using a quantitative measure of EOB. *Data Sources.* A systematic review of published sham RCTs that assessed blinding. *Study Selection.* Five hundred and ninety studies were reviewed, and 54 studies (4783 subjects) were included. *Data Extraction.* The number of patients who guessed their treatment identity was extracted from each study. Variables with possible influence on blinding were identified. *Data Synthesis.* The blinding index was calculated for each study. Based on blinding indexes, studies were congregated into one of the nine blinding scenarios. Individual study characteristics were explored for potential association with EOB. *Limitations.* There is a possibility of publication or reporting bias. *Conclusions.* The most common scenario was that the subjects believed they received verum acupuncture regardless of the actual treatment received, and overall the subject blinding in the acupuncture studies was satisfactory, with 61% of study participants maintaining ideal blinding. Objectively calculated blinding data may offer meaningful and systematic ways to further interpret the findings of RCTs.

## 1. Introduction

The presence of a viable control is important for any research study, allowing for valid comparison to the condition of interest. Randomized controlled trials (RCTs) are the primary way that many areas of research examine the comparative effectiveness of verum and control conditions. Patient blinding in these studies is essential for gathering reliable results that can be expanded upon. However, blinding success in nonpharmacologic interventions, such as acupuncture, can be difficult to assess, and results may not be applicable from one study to the next, due to differences in control methods and in how blinding success is determined, if it is considered at all. The CONSORT 2010 Statement on blinding suggests that it is important to include how blinding was attempted, but there is no mention in the statement of how to assess whether blinding was successful [1]. Without knowing if the specific nonpharmacologic blinding techniques used are valid overall, the information collected may or may not be reliable. We believe it is imperative that blinding success be assessed toward better understanding of the reliability of results.

With respect to acupuncture research specifically, developing a control procedure that is physiologically inert and indistinguishable from true treatment has proven to be a challenge due to the very nature of traditional acupuncture. It has resulted in a variety of control methods that up until now have not been compared in terms of blinding success [2–5]. The traditional techniques of acupuncture (penetrating needles at acupoints for different organ systems and deqi) are the verum conditions of most acupuncture studies. Control conditions include penetrating needles at “nonpoints” and “wrong points,” commercially-developed nonpenetrating devices, homemade nonpenetrating needles, and toothpicks or cocktail sticks [6–9]. “Nonpoints” are points not used for any purpose in traditional acupuncture. For the “wrong points,” sham acupuncture is done at points thought to affect a different body system than the one targeted by the verum condition. However, the “wrong point” method may cause a physiological effect similar to that of acupuncture and, therefore, may be more appropriately considered to be verum than sham acupuncture [10]. 

To date, there is no standard or universally accepted sham procedure for acupuncture research and no quantitative comparison of blinding between the above sham methods. This may contribute to why there is a discrepancy between the clinically recognized effectiveness of acupuncture and the relative lack of research supporting it [11]. Methodological progress for blinding characteristics, including the amount of disclosure to study participants, the variables to be collected, the analytic design, and the interpretation strategy, is needed in validation studies of sham control procedures [12].

The present meta-analysis systematically examines the status of blinding in sham acupuncture RCTs via a numerical measure of blinding index. Our primary aim is to empirically evaluate the validity (via effectiveness of blinding) of sham control techniques in order to quantitatively assess blinding across available studies. We hope to determine the reliability of the results of studies that used different sham techniques, so that we may learn which sham methods are most useful for future acupuncture research. We also believe that our systematic review is just the beginning of increasing the validity of the assessment of quantitatively assessed blinding practices in acupuncture research. Our methods can be further extended as a model to assess other nonpharmacologic treatments' blinding techniques.

## 2. Methods

### 2.1. Data Sources and Searches

PubMed, Embase, and Web of Knowledge databases were searched for scientific articles using the keywords “acupuncture,” “sham acupuncture,” or “sham procedure.” A revised search was also performed with Ovid Medline using the keywords “acupuncture,” “sham acupuncture,” or “placebo acupuncture.” Eligible studies were those that were randomized controlled in humans and were published in English between 1985 and 2011. Our search was not limited by patient diagnosis or by the part of the body where acupuncture was administered.

### 2.2. Study Selection

A study was considered eligible for initial screening if the authors stated that they evaluated blinding. One of the authors (Moroz, Freed, or Tiedemann) determined if the study reported data on effectiveness of blinding (EOB) by specifically asking participants if they thought the treatment used verum (V, real needle used or needle penetrated the skin) or sham (S) needling techniques, with or without an option to say they did not know (DK). If the blinding evaluation data was not included or was unclear, the authors were contacted twice by e-mail and/or telephone and asked for additional blinding evaluation data or clarification. Studies whose authors did not respond or responded but no longer had access to the original data were excluded. Studies that used a credibility questionnaire, asking patients to choose whether or not they had received treatment based on the principles of Chinese medicine or another type of acupuncture, were excluded as well. In these studies, the patients' ability to distinguish between verum and sham needling was not directly addressed and the questionnaire could be misinterpreted. If EOB was measured multiple times within the same study, the data collected at the end of the study or the data collected from the insertion (as opposed to sensation of deqi or needle withdrawal) arm of the study would be used. In one work that reported results of two separate studies using different acupuncture points, one on the patients' back and the other on the upper extremity, the blinding index calculations were done independently [58].

### 2.3. Data Extraction

For the studies that were included in this meta-analysis, the number of patients who responded as V, S, or DK was extracted from each trial. Additionally, the following variables were extracted from each of the included studies (we hypothesized a priori that these may be associated with EOB): year of publication, subject only or staff and subject blinding, time of blinding assessment, assessment of deqi, type of sham control device used, use of penetrating or nonpenetrating sham control, patient diagnosis, and number of days without acupuncture experience prior to participation. Research staff members that were blinded were those involved in data analysis and interpretation, not those administering acupuncture.

### 2.4. Data Synthesis and Analysis

Statistical analyses were carried out using the blinding index (BI) in order to objectively assess EOB [63, 64]. Blinding index estimates the degree of potential unblinding beyond chance for each arm in a given study by counting the excessive numbers of correct guesses. Blinding index values are always between −1 and 1, where 1 corresponds to all correct guesses, whereas −1 corresponds to all incorrect or opposite guesses. If 50% of patient responses are correct and 50% are incorrect, then BI = 0; this is indicative of random guessing and thus is an ideal blinding scenario. Another plausible scenario indicating effective blinding is that patients tend to believe they received active treatment regardless of actual treatment received, which may reflect patients' wish to receive active intervention. In this case, blinding index will have a positive value in the active treatment arm and a negative value in the sham treatment arm, where this scenario is denoted later as unblinded/opposite.

Verum and sham acupuncture groups were each assigned a separate blinding index value. Based on the calculated blinding index value combinations for the two treatment arms, nine possible blinding scenarios were proposed (Table 1). For classification purposes, we decided to consider BI ≥ 0.2 unblinded; −0.2 < BI < 0.2 random guesses; BI ≤ −0.2 opposite guesses [13, 64, 65]. (Remark: this cutoff value was based on authors' consensus and used as a general tool for classification and explanation; it should not be interpreted as an absolute indication of blinding effectiveness.)

Individual variables hypothesized to potentially impact EOB were compared by their average VBI and SBI scores, weighted by sample size. The weighted averages were used to determine the overall blinding index scenario for each variable. Blinding scenarios were also compared to the overall outcome of each study. Finally, we looked for patterns of possible association of study design factors with EOB based on blinding index values and scenarios. The factors included were based on data extraction criteria, sample size, timing of blinding assessment, blinded parties, sensation assessed, subject's status, subject's experience, and sham device used.

## 3. Results

### 3.1. Data Search

**Figure 1:**
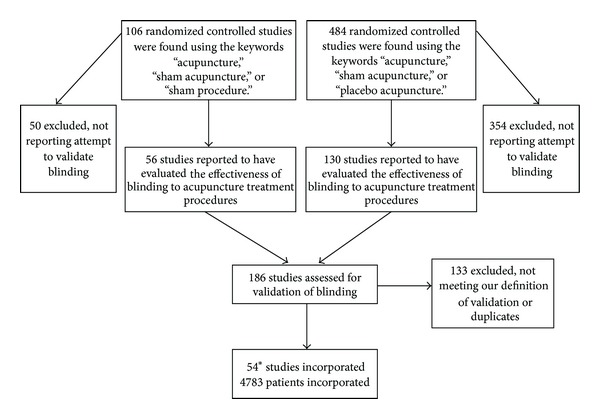
Systematic review search and selection. *One article contained two separate studies.

Using our search inclusion criteria, 590 peer reviewed journal articles were found, with 186 of these reporting blinding data in RCTs. 133 studies were excluded from the review, most often due to a lack of patient guess of treatment allocation. One article reported two distinct studies that were included separately [58]. Fifty-four studies were included in our final analysis, with a total of 4783 patients (Figure 1).

### 3.2. Blinding Index Calculations

**Figure 2:**
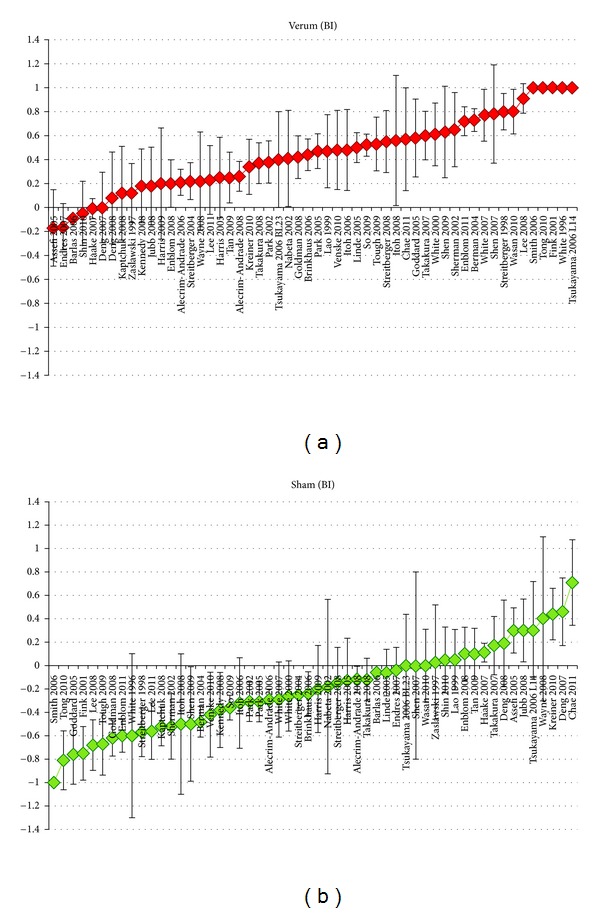
(a) Verum and (b) sham. Blinding index values with 95% confidence intervals. *Individual blinding index estimate and confidence intervals raw data are provided in Table 3. Confidence intervals are unadjusted for multiple comparisons.

The blinding index values (point and interval estimates) computed from all 54 studies can be found in Figure 2. The average weighted blinding index values for the entire review were 0.34 for verum and −0.20 for sham groups, respectively. Overall, a correct guess is quite common in the verum arm, and opposite guess is not uncommon in the sham arm.

After grouping studies into the nine possible blinding scenarios based on the blinding index (Table 1), 33 out of 54 (61%) of the studies might be adequately blinded. Of these, 70% (23/33) had a positive outcome reported overall; similarly, 62% (13/21) with less ideal blinding had an overall positive outcome (Table 3). Unblinded/opposite for V, S is most common, with 46% of the studies belonging to this scenario, followed by unblinded/random with 22%.

### 3.3. Design Characteristics and Effectiveness of Blinding

The variables hypothesized to affect blinding were compared by their average VBI and SBI values, and blinding scenarios in Table 2.

Of the 54 studies, 22 studies used a commercially developed sham control device, 14 studies used a custom-made sham control device, 12 used penetrating sham control, and 6 used a toothpick or cocktail stick. According to their averaged blinding index scenarios, all of the sham control devices with the exception of custom devices seemed to be effective in blinding the subjects, with the penetrating sham controls providing relatively more effective blinding.

In looking at the penetrating versus nonpenetrating dichotomy in a more direct way, the nonpenetrating group was unblinded/opposite, the mixed penetrating/nonpenetrating group was unblinded/opposite, and the penetrating group was random/random. All of these scenarios indicated effective blinding.

Studies with a greater number of subjects had a greater unblinding in the verum group. Measurements assessed later tended to have more ideal effectiveness of blinding than measurements assessed immediately. Interestingly, there was a higher tendency in both arms, when only the subjects—not staff—were blinded, to believe they received the verum treatment. The deqi group had slightly better EOB than the insertion/puncture group, and the symptomatic group had more ideal EOB than the healthy group.

Twenty-four studies used acupuncture-naïve subjects, and 19 used subjects with prior acupuncture experience. Eleven of the studies were excluded from this section of the review due to unknown prior experience or mixed experience of the subjects. According to their averaged blinding index scenarios, both groups were unblinded/random.

## 4. Discussion

This systematic review of 54 randomized controlled acupuncture studies showed that overall 61% of the studies (as a conservative estimate) meeting our inclusion/exclusion criteria were effectively blinded. The most common scenario encountered was unblinded in the acupuncture group and opposite guess in the sham acupuncture group, which could be indeed interpreted as “well-blinded.” In this scenario there may be a psychological phenomenon of “wishful thinking.” A majority of people, in both the verum and sham groups, guessed that they received real acupuncture. Thus, guesses are inflated towards real acupuncture in both study arms. It is also possible that once a needle is administered, a subject believes it is real acupuncture, or subjects may not know what to expect, creating a similar trend, or there is a strong placebo effect.

 A similar pattern emerges when looking at the V and S groups individually; the average VBI was “unblinded” and the average SBI was “opposite.” Is it possible for one not to know when a needle is penetrating his or her skin? Perhaps the answer is “no,” given that unblinded V may mean that subjects know when their skin is being penetrated by a needle and thus increases the chance a subject chooses the V acupuncture group over the S acupuncture group upon questioning. Is it possible for one to know when a needle is not penetrating their skin? The answer seems to be “no” again; opposite guesses in S may indicate that subjects are not able to tell if they are not being penetrated by a needle, and thus are truly guessing.

Most sham control devices with the exception of custom devices were effective in blinding the subjects (Table 2). Since there was a great diversity within the custom sham group a more in depth case by case analysis of each device could be performed, but at this time there does not seem to be compelling evidence supporting the use of custom sham devices. Even though commercial sham devices and toothpick/cocktail stick devices appear to provide effective blinding, the penetrating sham controls provided relatively more effective blinding. 

By comparing study blinding and study outcomes, the majority of studies reported positive outcomes, regardless of the degree of guess correctness. This leads us to believe that there is no obvious association between EOB and reported study outcomes. The current literature provides conflicting evidence so the direction of bias may be specific to context or treatment [6–9, 14–49, 51–62] or random.

Exploration of individual variables and their possible effect on EOB indicated that some design characteristics such as larger sample size, symptomatic subjects, and later assessment were associated with more effective blinding, and these may be encouraged to be further evaluated and considered in designing future acupuncture trials.

### 4.1. Recommendations for Future Acupuncture Research

The effect size was not a part of the extracted information from the reviewed studies. This poses an interesting idea for future investigation. Additional future research should include a sufficiently powered, prospective randomized trial comparing the EOB of different methods and sham devices by direct comparison, as well as investigating the influence of practitioner behavior, the patient's expectations and beliefs, and the location of treatment points on blinding effectiveness. A good treatment should have a greater treatment effect than placebo effect. It is important that we collect more data in this field, especially qualitative data (e.g., reasons of guessing in a particular way). It is possible that the reasons for correct guesses in individual trials may be more revealing than our numbers. Individual trialists should be willing to share their experiences with others, as individual trialists and patients must have greater insight and more stories to tell in specific conditions within the trial than meta-analysis and readers. This is particularly important, because any analysis of available numeric data on blinding is destined to be prone to some biases.

### 4.2. Systematic Review Limitations

There are several potential weaknesses of this review that we recognize. Language bias may be a possibility given that we included only English language publications while acupuncture is a popular treatment modality in Asia and Europe. There is also a possibility of publication bias, which is an inherent problem in virtually all systematic reviews or meta-analyses. Moreover, it is possible that some investigators collected blinding data, saw the data, and decided not to report it in the paper, particularly when blinding was shown to be unsatisfactory. Also, for classification and decision making purposes, we used categorization/dichotomization (e.g., numerical BI cutoff of 0.2) and summary statistics (e.g., average BI). Alternative ways of analyzing the data could yield different results. Finally, in this systematic review, we used the conventional terms commonly used in the literature and equated “unblinded” with “correctly guessed,” whereas there could be other reasons for correct guesses. With its limitations, to our knowledge, this is the first systematic review based on empirical blinding data.

In conclusion, based on the status of blinding, the most common scenario encountered was a more correct guess in the real acupuncture group and an opposite guess in the sham acupuncture group, and the overall subject blinding of the evaluated acupuncture studies was satisfactory. In addition, quantitatively calculated blinding data, ideally together with more qualitative data for individual trials, may offer meaningful means to further interpret the findings of RCTs and improve the practice in the direction of a higher validity. 

## Figures and Tables

**Table 1:** Blinding scenarios [13].

Experimental arm (verum)	Control arm (sham)	Possible blinding and clinical effectiveness interpretations	Trials number (%)
Random guess	Random guess	Ideal, possibly most ideal from the scientific or statistical perspective	8 (15)
Random guess	Opposite guess	Rare	2 (4)
Random guess	Unblinded	Possibly little treatment effect and completely no effect in control arm	3 (6)
Unblinded	Unblinded	Possibly problematic. Treatment effect in experimental arm and no treatment effect in control arm (e.g., patients tend to know what to expect)	4 (7)
Unblinded	Opposite guess	Ideal (e.g., patients tend to have wishful thinking, strong placebo effect, and any treatment administered is perceived as real treatment)	25 (46)
Unblinded	Random guess	Possibly problematic. Treatment effect in experimental arm and no treatment effect in control arm (e.g., patients do not know what to expect in the absence of treatment)	12 (22)
Opposite guess	Opposite guess	Rare	0 (0)
Opposite guess	Random guess	Rare	0 (0)
Opposite guess	Unblinded	No treatment effect at all; patients may have low expectations	0 (0)

**Table 2:** Design characteristics that may be associated with blinding, compared by BI values and scenarios.

Study characteristics	Number of studies	BI (V)	BI (S)	BI (|V| − |S|)	Blinding scenario
Sample size					
*N* < 100	43	0.44	−0.19	0.25	Unblinded/random
*N* ≥ 100	11	0.28	−0.20	0.08	Unblinded/opposite
Blinding assessed					
Immediately	19	0.50	−0.19	0.31	Unblinded/random
Later	35	0.29	−0.20	0.09	Unblinded/opposite
Blinded parties					
Subjects	22	0.52	−0.27	0.25	Unblinded/opposite
Subjects + research staff	32	0.26	−0.17	0.09	Unblinded/random
Assessed for sensation of					
Deqi	22	0.33	−0.21	0.12	Unblinded/opposite
Puncture	32	0.35	−0.19	0.16	Unblinded/random
Subject's status					
Healthy	12	0.43	−0.06	0.37	Unblinded/random
Symptomatic	42	0.33	−0.22	0.11	Unblinded/opposite
Subject's acupuncture experience					
Naïve	24	0.27	−0.13	0.14	Unblinded/random
Experienced	19	0.32	−0.19	0.13	Unblinded/random
Sham device used					
Commercial	22	0.47	−0.32	0.15	Unblinded/opposite
Custom	14	0.48	−0.17	0.31	Unblinded/random
Penetrating	12	0.16	−0.08	0.08	random/random
Toothpick or cocktail stick	6	0.55	−0.33	0.22	Unblinded/opposite

*Raw data available in Table 4.

**Table 3:** Blinding index values computed from 54 studies.

Study	*N*	VBI	VBI 95% CI	SBI	SBI 95% CI	Scenario	Study outcome
Assefi et al. 2005 [14]	75	−0.17	−0.49 to 0.15	0.3	0.10 to 0.49	Random/unblinded	Negative
Kaptchuk et al. 2008 [15]	148	0.12	−0.07 to 0.31	−0.52	−0.69 to −0.35	Random/opposite	Positive
Kennedy et al. 2008 [9]	45	0.18	−0.13 to 0.49	−0.38	−0.70 to −0.05	Random/opposite	Positive
Barlas et al. 2006 [6]	48	−0.09	NA	−0.06	NA	Random/random	Positive
Deng et al. 2008 [16]	53	0.08	−0.30 to 0.46	0.19	−0.18 to 0.55	Random/random	Negative
Enblom et al. 2008 [7]	80	0.2	0.00 to 0.40	0.1	−0.13 to 0.33	Random/random	Positive
Endres et al. 2007 [17]	403	−0.16	−0.35 to 0.03	−0.04	−0.24 to 0.16	Random/random	Positive
Haake et al. 2007 [8]	692	−0.01	−0.09 to 0.07	0.11	0.03 to 0.19	Random/random	Positive
Harris et al. 2009 [18]	20	0.2	−0.26 to 0.66	−0.2	−0.57 to 0.17	Random/random	Positive
Shin et al. 2010 [19]	42	−0.05	−0.35 to 0.18	0.05	−0.23 to 0.33	Random/random	Negative
Zaslawski et al. 1997 [20]	64	0.12	−0.37 to 0.13	0.03	−0.28 to 0.21	Random/random	Positive
Deng et al. 2007 [21]	67	0	−0.29 to 0.29	0.46	0.17 to 0.75	Random/unblinded	Negative
Jubb et al. 2008 [22]	51	0.18	−0.14 to 0.51	0.3	0.03 to 0.56	Random/unblinded	Positive
Alecrim-Andrade et al. 2006 [23]	24	0.21	0.11 to 0.33	−0.29	−0.37 to −0.20	Unblinded/opposite	Negative
Berman et al. 2004 [24]	283	0.73	0.63 to 0.82	−0.48	−0.61 to −0.35	Unblinded/opposite	Positive
Brinkhaus et al. 2006 [25]	205	0.44	0.30 to 0.57	−0.24	−0.42 to −0.05	Unblinded/opposite	Positive
Enblom et al. 2011 [26]	190	0.72	0.60 to 0.83	−0.6	−0.74 to −0.46	Unblinded/opposite	Positive
Fink et al. 2001 [27]	64	1	NA	−0.75	−0.98 to −0.52	Unblinded/opposite	Positive
Goddard et al. 2005 [28]	49	0.58	0.26 to 0.91	−0.76	−1.01 to −0.50	Unblinded/opposite	Positive
Goldman et al. 2008 [29]	118	0.42	0.24 to 0.60	−0.62	−0.77 to −0.47	Unblinded/opposite	Negative
Itoh et al. 2006 [30]	19	0.48	0.14 to 0.81	−0.32	−0.70 to 0.07	Unblinded/opposite	Positive
Itoh et al. 2008 [31]	24	0.56	0.01 to 1.10	−0.5	−1.10 to 0.10	Unblinded/opposite	Positive
Lee et al. 2008 [32]	89	0.91	0.79 to 1.03	−0.68	−0.90 to −0.47	Unblinded/opposite	Positive
Lee et al. 2011 [33]	35	0.23	−0.06 to 0.51	−0.56	−0.80 to −0.31	Unblinded/opposite	Positive
Park et al. 2002 [34]	58	0.38	0.20 to 0.56	−0.31	−0.48 to −0.14	Unblinded/opposite	Positive
Park et al. 2005 [35]	94	0.47	0.32 to 0.61	−0.31	−0.48 to −0.13	Unblinded/opposite	Negative
Shen et al. 2009 [36]	12	0.63	0.24 to 1.01	−0.5	−0.99 to −0.01	Unblinded/opposite	Positive
Sherman et al. 2002 [37]	52	0.65	0.34 to 0.96	−0.52	−0.80 to −0.24	Unblinded/opposite	Positive
Smith et al. 2007 [38]	27	1	NA	−1	NA	Unblinded/opposite	Positive
So et al. 2009 [39]	370	0.52	0.43 to 0.61	−0.36	−0.46 to −0.25	Unblinded/opposite	Negative
Streitberger and Kleinhenz 1998 [40]	60	0.8	0.65 to 0.95	−0.57	−0.77 to −0.36	Unblinded/opposite	Positive
Streitberger et al. 2004 [41]	212	0.22	0.06 to 0.37	−0.25	−0.38 to −0.11	Unblinded/opposite	Negative
Tong et al. 2010 [42]	63	1	NA	−0.81	−1.06 to −0.56	Unblinded/opposite	Positive
Tough et al. 2009 [43]	37	0.57	0.30 to 0.75	−0.67	−0.93 to −0.40	Unblinded/opposite	Positive
Venzke et al. 2010 [44]	51	0.48	0.15 to 0.81	−0.42	−0.78 to −0.05	Unblinded/opposite	Negative
White et al. 1996 [45]	9	1	NA	−0.6	−1.30 to 0.10	Unblinded/opposite	Negative
White et al. 2000 [46]	44	0.61	0.35 to 0.87	−0.26	−0.56 to 0.04	Unblinded/opposite	Negative
White et al. 2007 [47]	37	0.77	0.55 to 0.98	−0.29	−0.61 to 0.03	Unblinded/opposite	Positive
Alecrim-Andrade et al. 2008 [48]	36	0.26	0.14 to 0.39	−0.12	−0.23 to −0.00	Unblinded/random	Negative
Harris et al. 2005 [49]	76	0.25	−0.09 to 0.59	−0.13	−0.50 to 0.23	Unblinded/random	Negative
Lao et al. 1999 [50]	39	0.47	0.17 to 0.78	0.05	−0.21 to 0.31	Unblinded/random	Positive
Linde et al. 2005 [51]	201	0.5	0.38 to 0.63	−0.06	−0.26 to 0.15	Unblinded/random	Negative
Nabeta and Kawakita 2002 [52]	34	0.41	0.01 to 0.81	−0.18	−0.61 to 0.26	Unblinded/random	Positive
Shen and Goddard 2007 [53]	15	0.78	0.37 to 1.19	0	−0.80 to 0.80	Unblinded/random	Positive
Streitberger et al. 2008 [54]	20	0.55	0.29 to 0.81	−0.15	0.46 to 0.16	Unblinded/random	Positive
Takakura and Yajima 2007 [55]	60	0.6	0.40 to 0.80	0.17	−0.08 to 0.42	Unblinded/random	Positive
Takakura and Yajima 2008 [56]	114	0.37	0.20 to 0.54	−0.12	−0.30 to 0.06	Unblinded/random	Positive
Tan et al. 2009 [57]	20	0.25	0.04 to 0.46	0.1	−0.12 to 0.32	Unblinded/random	Positive
Tsukayama et al. 2006 BL23 [58]	20	0.4	−0.00 to 0.80	0	−0.44 to 0.44	Unblinded/random	Negative
Wasan et al. 2010 [59]	40	0.8	0.61 to 0.99	0	−0.31 to 0.31	Unblinded/random	Negative
Chae et al. 2011 [60]	28	0.57	0.14 to 1.00	0.71	0.35 to 1.08	Unblinded/unblinded	Positive
Kreiner et al. 2010 [61]	32	0.34	0.11 to 0.57	0.44	0.22 to 0.66	Unblinded/unblinded	Positive
Tsukayama et al. 2006 L14 [58]	20	1	NA	0.3	−0.12 to 0.72	Unblinded/unblinded	Negative
Wayne et al. 2008 [62]	14	0.22	−0.19 to 0.63	0.4	−0.30 to 1.10	Unblinded/unblinded	Positive

**Table 4:** Study characteristics extracted from 54 studies.

Study	Total *N*	Party blinded	Time of blinding assessment (days from beginning of study)	Deqi assessment (yES/nO)	Sham device	Penetration of sham	Study participant diagnosis	Days since last acupuncture experience
Shen and Goddard 2007 [53]	15	Staff + subject	0	NO	C	NP	Chronic TM pain	Unknown
Itoh et al. 2006 [30]	19	Staff + subject	21	NO	C	NP	Chronic LBP	Unknown
Nabeta and Kawakita 2002 [52]	34	Subject	28	NO	C	NP	Chronic neck and shoulder pain	Unknown
Tough et al. 2009 [43]	37	Subject	14	NO	C	NP	Whiplash	Unknown
Fink et al. 2001 [27]	64	Staff + subject	14	NO	C	NP	Tension headache	Unknown
Shen et al. 2009 [36]	12	Staff + subject	0	YES	C	NP	Chronic myofacial pain of jaw muscles	>365
Kreiner et al. 2010 [61]	32	Staff + subject	0	NO	C	NP	Normal	>365
Lao et al. 1999 [50]	39	Staff + subject	0.2	YES	C	NP	After oral surgery Pain	>365
Goddard et al. 2005 [28]	49	Subject	0	YES	C	NP	Normal	>365
Jubb et al. 2008 [22]	51	Subject	63	YES	C	NP	Knee OA	>365
Itoh et al. 2008 [31]	24	Staff + subject	35	NO	C	NP	Knee OA	180
Deng et al. 2008 [16]	53	Subject	30	NO	C	NP	After thoracotomy Pain	42
Takakura and Yajima 2007 [55]	60	Staff + subject	0	NO	C	NP	Normal	0
Takakura and Yajima 2008 [56]	114	Staff + subject	0	YES	C	NP	Normal	0
Smith et al. 2007 [38]	27	Staff + subject	28	NO	P	NP	TMJ	Unknown
Tan et al. 2009 [57]	20	Subject	0	NO	P	NP	Normal	>365
Kennedy et al. 2008 [9]	45	Subject	42	NO	P	NP	Acute LBP	>365
Park et al. 2002 [34]	58	Staff + subject	84	NO	P	NP	Acute stroke	>365
Park et al. 2005 [35]	94	Staff + subject	14	NO	P	NP	Stroke	>365
Enblom et al. 2011 [26]	190	Staff + subject	35	NO	P	NP	Radiation-induced nausea	365
Tsukayama et al. 2006 BL23 [58]	20	Staff + subject	0	NO	P	NP	Normal	0
Tsukayama et al. 2006 L14 [58]	20	Staff + subject	0	NO	P	NP	Normal	0
Chae et al. 2011 [60]	28	Subject	0	NO	P	NP	Normal	0
Shin et al. 2010 [19]	42	Staff + subject	21	YES	PEN	P	Dry eye	Unknown
Tong et al. 2010 [42]	63	Subject	15	YES	PEN	P	DPN	~50%
Zaslawski et al. 1997 [20]	64	Subject	28	YES	PEN	P	Stress	~50%
Harris et al. 2005 [49]	76	Staff + subject	21	NO	PEN	P	Fibromyaligia	>365
Haake et al. 2007 [8]	692	Staff + subject	168	NO	PEN	P	Chronic LBP	>365
Linde et al. 2005 [51]	201	Staff + subject	168	YES	PEN	P	Migraine HA	365
Brinkhaus et al. 2006 [25]	205	Subject	56	YES	PEN	P	Chronic LBP	365
Endres et al. 2007 [17]	403	Staff + subject	180	YES	PEN	P	Tension headache	365
Alecrim-Andrade et al. 2006 [23]	24	Staff + subject	84	YES	PEN	P	Migraine HA	84
Alecrim-Andrade et al. 2008 [48]	36	Subject	84	YES	PEN	P	Migraine HA	84
Lee et al. 2011 [33]	35	Staff + subject	70	NO	PEN	P	Chronic pelvic pain	42
Lee et al. 2008 [32]	89	Subject	70	NO	PEN	P	Chronic prostatitis	42
White et al. 2007 [47]	37	Subject	0	YES	S	NP	Chronic Pain/Normal	Unknown
So et al. 2009 [39]	370	Staff + subject	0	NO	S	NP	Infertility	Unknown
Wayne et al. 2008 [62]	14	Staff + subject	28	NO	S	NP	Endometriosis	>365
Streitberger et al. 2008 [54]	20	Subject	0	NO	S	NP	Normal	>365
Wasan et al. 2010 [59]	40	Subject	0	YES	S	NP	Chronic LBP	>365
Barlas et al. 2006 [6]	48	Staff + subject	0	YES	S	NP	Normal	>365
Streitberger and Kleinhenz 1998 [40]	60	Subject	0	NO	S	NP	Normal	>365
Enblom et al. 2008 [7]	80	Subject	0	NO	S	NP	Normal	>365
Kaptchuk et al. 2008 [15]	148	Staff + subject	42	NO	S	NP	IBS	>365
Goldman et al. 2008 [29]	118	Staff + subject	28	YES	S	NP	Arm pain	365
Venzke et al. 2010 [44]	51	Subject	168	YES	S	NP	Hot flashes	180
Streitberger et al. 2004 [41]	212	Staff + subject	1	YES	S	NP	postoperative nausea	180
Deng et al. 2007 [21]	67	Subject	42	YES	S	NP	Hot flashes	42
White et al. 1996 [45]	9	Staff + subject	42	NO	T	NP	Tension headache	>365
Harris et al. 2009 [18]	20	Staff + subject	0	NO	T	NP	Fibromyalgia	>365
White et al. 2000 [46]	44	Staff + subject	7	YES	T	NP	Tension headache	>365
Sherman et al. 2002 [37]	52	Subject	0	NO	T	NP	Chronic LBP	>365
Assefi et al. 2005 [14]	75	Staff + subject	84	NO	T	NP, P	Fibromyalgia	>365
Berman et al. 2004 [24]	283	Subject	182	YES	T	NP, P	Knee OA	>365

Sham devices: C: custom sham, P: park sham, PEN: penetrating sham, S: streitberger sham, and T: toothpick.
